# Preparation of Fouling-Resistant Nanofibrous Composite Membranes for Separation of Oily Wastewater

**DOI:** 10.3390/polym9120679

**Published:** 2017-12-06

**Authors:** Fatma Yalcinkaya, Anna Siekierka, Marek Bryjak

**Affiliations:** 1 Department of Nanotechnology and Informatics, Technical University of Liberec, Institute for Nanomaterials, Advanced Technologies and Innovation, Studentska 1402/2, 46117 Liberec, Czech Republic; 2 Faculty of Chemistry, Wroclaw University of Science and Technology, 27 Wybrzeze Stanislawa Wyspianskiego, 50-370 Wroclaw, Poland; anna.siekierka@pwr.edu.pl (A.S.); marek.bryjak@pwr.edu.pl (M.B.)

**Keywords:** nanofiber, modified nanofiber, nanocomposite

## Abstract

A facile and low-cost method has been developed for separation of oily wastewater. Polyvinylidene fluoride/polyacrylonitrile (PVDF/PAN) nanofibers laminated on a supporting layer were tested. In order to create highly permeable and fouling-resistant membranes, surface modifications of both fibers were conducted. The results of oily wastewater separation showed that, after low vacuum microwave plasma treatment with Argon (Ar) and chemical modification with sodium hydroxide (NaOH), the membranes had excellent hydrophilicity, due to the formation of active carboxylic groups. However, the membrane performance failed during the cleaning procedures. Titanium dioxide (TiO_2_) was grafted onto the surface of membranes to give them highly permeable and fouling-resistance properties. The results of the self-cleaning experiment indicated that grafting of TiO_2_ on the surface of the membranes after their pre-treatment with Ar plasma and NaOH increased the permeability and the anti-fouling properties. A new surface modification method using a combination of plasma and chemical treatment was introduced.

## 1. Introduction

The increasing amount of industrial and domestic oily wastewater has become one of the most important problematic issues for the environment and human health. Oil–water emulsion separation has gained more importance in recent decades. A few methods have been developed for oil–water separation, such as oil containment booms [1], coagulation method [2], oil sorption materials [3,4,5], oil skimmers [1], air flotation [6], and combustion [7]. However, these methods have disadvantages, like secondary pollution, expensive operation, low efficiency, complicated operation, and they are time-consuming. Microfiltration is one of the simplest and widely used methods for separation of oily wastewater. Many researchers have developed various types of microfilters. Membranes are the most important part of the microfiltration process [8]. For instance, it was found that superhydrophilic in situ-crosslinked zwitterionic polyelectrolyte/polyvinylidene fluoride-blend membranes exhibit high water permeation flux and good antifouling properties for separating oil-in-water emulsions with high separation efficiency [9]. Cao et al. prepared hyperbranched polyethyleneimine (HPEI) glass fiber membranes for oil–water separation. The modified membrane showed high permeation and quantitative oil rejection with excellent thermal and chemical stability, compared with polymer-based membranes [10]. Cumming et al. [11] developed a method for characterizing the rejection efficiency, by using an asymmetric metal microfilter to separate oil in a water dispersion. Results showed that the rejection of oil drops depended on the size distribution of the emulsion, and the use of a surface filter without any internal tortuosity excluded the possibility of internal fouling. However, fouling is a common problem that has to be faced during microfiltration. In fouling, the membrane is contaminated by a solution or particle, which results in a decrease in membrane performance. In general, fouling forms on hydrophobic surfaces as a result of aggregation, protein adsorption, and denaturation at the membrane–solution interface [12].

Fouling causes a decrease in the performance of the filters, and generates extra costs for repetitive cleaning procedures. For effective oily water treatment, antifouling membranes with very high selectivity are required. More often, the selectivity and the fouling resistant properties of the membranes are strongly dependent on their surface wettability. With this aim in mind, many researchers have employed various modifications to change the surface properties of the polymeric membranes, such as surface grafting, blending, surface coating, and surface absorption [13,14,15,16,17,18,19]. Zhang et al. [20] fabricated a Graphene oxide modified polyacrylonitrile hierarchical-structured membrane. It was found that this hierarchical-structured membrane exhibited a very high flux, feasible rejection ratio, and superior antifouling performance in separating an oil–water emulsion, due to its surface hydrophilicity. In another work [21], pancreatic enzyme was immobilized on polyethersulfone membranes by electron beam modification. The anti-fouling property of the membrane was obtained after switching on the catalytic activity of the enzyme by adjusting the pH and temperature. As a result, the membrane surface actively degraded a fouling layer, and regained its initial permeability. Yang et al. [22] fabricated a superhydrophilic and superoleophobic nanocomposite coating by spray casting nanoparticle–polymer suspensions on various substrates. They synthesized the polymer with hydrophilic and oleophobic properties by using the reaction of poly (diallyldimethylammonium chloride) (PDDA) with sodium perfluorooctanoate (PFO) in aqueous solution, in which PFO anions can coordinate to quaternary ammonium groups of PDDA. As a result of the high surface concentration of fluorinated groups, together with carboxyl and quaternary ammonium groups, oleophobic and hydrophilic material were fabricated. Water molecules are able to penetrate the surfaces, while oils cannot. An air plasma treatment was applied to enhance the hydrophilicity of the coating material and increase the water permeability, while there was no change in superoleophobic properties. Wei et al. [23] used maleic anhydride to graft onto a polyacrylonitrile (PAN) membrane surface via ultraviolet irradiation. Hyperbranched polyester grafting onto the PAN membrane surface, by the reaction of hydroxyl groups with anhydride groups of maleic anhydride, followed the grafting process. The filtration showed that modified membranes had a 4–6 times higher water flux and better antifouling properties than pristine PAN membranes, and their hydrophilicity was significantly improved. Zhang et al. [14] fabricated an ultralow oil-fouling amphiphilic copolymer incorporated poly(ether sulfone) (PES) heterogeneous membrane. First, the amphiphilic copolymer was prepared by semibatch reversible addition—where the fragmentation chain was transferred by poly (ethylene glycol) methyl ether methacrylate (PEGMA) and 3,3,4,4,5,5,6,6,7,7,8,8,8-tridecafluorooctyl acrylate (TFOA). The resultant amphiphilic fluorinated gradient copolymers were then incorporated into the PES. The resultant membrane showed effective oil–water emulsion separation due to the hydrophilicity of PEG and low surface energy of PTFOA. Wang et al. [24] prepared titanium dioxide (TiO_2_) doped polyvinylidene fluoride (PVDF) nanofibers to prevent fouling of membranes in oil–water separation. TiO_2_ gel was prepared and mixed with the PVDF solution before the spinning process. The membranes showed reversible separation of the oily water by UV (or sunlight) irradiation and heating treatment. Among various materials, TiO_2_ has been widely used, due to its self-cleaning and photocatalytic properties [25,26].

In this work, nanofibers were produced as an active layer for microfiltration. The properties of the nanofibers, such as their ability to be embedded within other media, high surface-to-volume ratio, large porosity, narrow pore size, easiness to operate, and adjustable functionality, are much more effective than conventional polymeric membranes used in liquid filtration. Nanofibers have a porosity of over 80% in the structure, which improves the filtration efficiency of the membranes. The low mechanical strength of the nanofibers restricts their application in liquid filtration [27]. To overcome this problem, a special lamination technique was applied, and nanofibrous composite membranes were formed as microfilters. The principle of the lamination technique has been explained in our previous work [28,29,30]. The use of this lamination technique did not change the properties of the nanofibers on the surface of the membrane. A polyester nonwoven layer was selected as a supporting layer for the nanofibers. The lamination technology provides excellent adhesion of the nanofibers to the substrate, as well as durable structural stability, which provides a longer lifespan and greater effectiveness in the cleaning process. In this work, a mixture of PVDF and polyacrylonitrile (PAN) nanofibers were fabricated. PVDF has a high hydrophobicity and thermal stability, good chemical resistance and oleophilicity. Its good electrical properties result from the polarity of alternating groups on the polymer chain, and easiness to fabricate the nanofiber web [31,32]. PAN has good characteristics, including thermal stability, tolerance to most solvents, and commercial availability [33]. PAN nanofibers are more hydrophilic and better at plasma etching compared to PVDF [34,35]. However, PAN nanofibers have lower mechanical and abrasion resistance than PVDF. The aim of mixing both polymers is to improve the mechanical properties of the nanofiber web, while providing an effective plasma treatment. In addition, both of the polymers were selected due to their relative low cost and widespread commercial use.

The selectivity and permeability are two key factors in the membrane process. The selectivity of membranes is largely determined by the surface porosity and pore size of the substructure, and the chemical and physical properties of the membrane, while the permeability mainly depends on the hydrophilicity, porosity, and pore size of the membrane. Due to the lack of functional groups on the PVDF/PAN nanofibrous membrane surface, it is necessary to introduce some functional groups by surface modification. In this study, the nanofibrous composite membranes were covered by TiO_2_ nanoparticles, due to their high stability, high photocatalytic activity, non-toxicity, low cost, chemical resistance, and antibacterial activity to certain microorganisms. TiO_2_ nanoparticles can be used to successfully overcome the fouling problem. The nanoparticles were grafted on the surface of nanofibrous membranes using plasma and chemical pre-treatments. According to our knowledge, this method has not been reported so far. The ultimate goal of this work was to introduce a new surface modification method that could offer highly permeable and fouling resistant membranes.

## 2. Materials and Methods

### 2.1. Preparation of the Nanofiber Web

**Figure 1:**
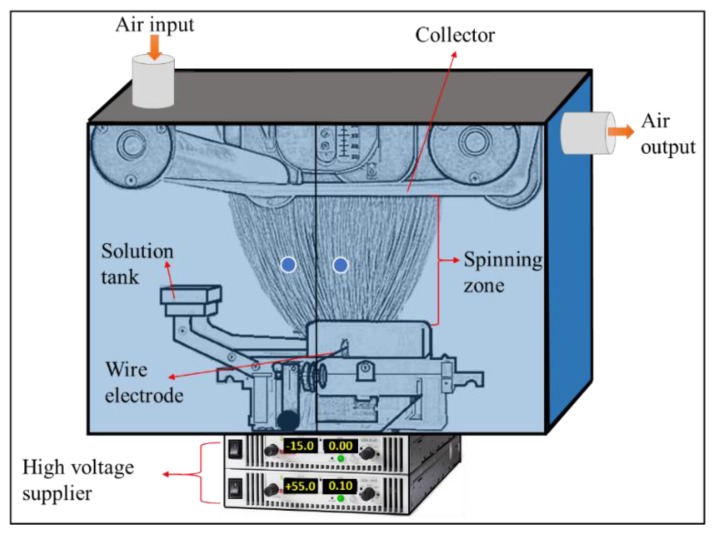
Schematic diagram of the Nanospider device.

A total of 8 wt % polyacrylonitrile (PAN) (*M*_W_ = 150 kDa, purchased from Elmarcos.r.o., Liberec, Czech Republic) was dissolved in dimethylformamide (DMF), while 13 wt % polyvinylidene fluoride (PVDF) (form from Solef 1015, Bruxelles, Belgium) was dissolved in dimethylacetamide (DMAc). Solvents were purchased from Penta, s.r.o., Prague, Czech Republic. The solutions were stirred overnight. A nanofiber web blend was prepared. The blend ratio of PVDF/PAN nanofibers in the composite was 1/2 in wt %. This ratio was determined based on our previous experience [34,36]. A lab-scale Nanospider (Elmarco s.r.o., Liberec, Czech Republic) electrospinning device was used for the production of nanofibers under stable conditions (Figure 1). A solution tank fed the solution toward the wire electrode. If the electrical fields between the wire electrode and the collector overcome the surface tension, Taylor’s cones were formed, and jets moved towards a take-up cylinder connected to a supporting material. The spinning conditions were kept stable by controlling the humidity, temperature and air input–output speed.

### 2.2. Formation of a Nanofibrous Composite Membrane

**Figure 2:**
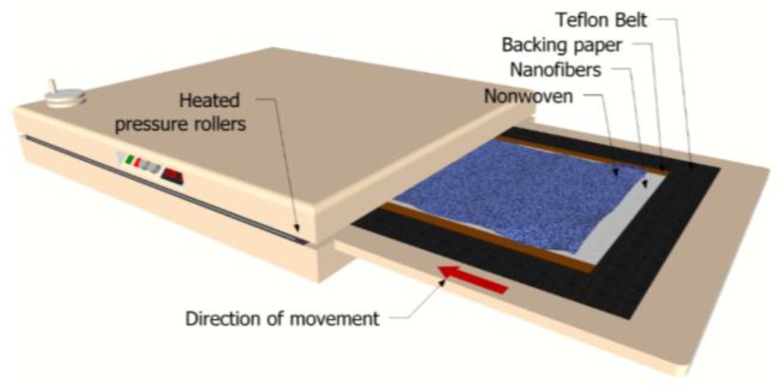
Lamination method and equipment [37].

The nanofiber web was gently laminated on a supporting layer using a Meyer RPS-L Mini lamination machine (Maschinenfabrik Herbert Meyer GmbH, Roetz, Germany) at room temperature (Figure 2).

The nanofiber web was collected on silicon paper. A 100 g/m^2^ polyethylene terephthalate spunbond nonwoven fabric was used as supporting layer, and 3 g/m^2^ of co-polyamide adhesive web were used to adhere the nanofibers and the nonwoven web. The lamination machine had a conveyer belt resistant to heat and damage. The maximum width of the samples was set as 400 mm, while there was no limitation on the length. The configuration of the substrate mainly depended on the application, and could be varied to reach the desired structural properties, including strength, stiffness, and durability, pliability and flexibility, and temperature resistance.

The zero-shear viscosity of the polymer solutions was obtained using a Fungilab Expert viscometer (Fungilab Leading Viscosity Technology, Barcelona, Spain) at 23 °C. The surface of the membranes was characterized using a Scanning Electron Microscope (SEM, Vega 3SB, Brno, Czech Republic) and fiber diameter, diameter distribution, and porosity were analyzed using the Image-J program (free online program). The surface contact angle of the samples was measured at room temperature using a Kruss Drop Shape Analyzer DS4 (Kruss GmbH, Hamburg, Germany), using distilled water on the clean and dry sample. An 1200-AEL capillary flow porometer (Porous Media Inc., Ithaca, NY, USA) was used in this study to measure the pore size. FTIR spectra were used to verify the effect of the plasma and chemical modifications on the composite membrane surface. The polymeric nanofiber membranes were evaluated using Fourier transform infrared spectroscopy (FTIR, Nicolet iZ10 by Thermo Fisher Scientific, Waltham, MA, USA).

### 2.3. Surface Treatment

The membranes were then subjected to the low vacuum plasma treatment described in the literature [38]. Microwave plasma treatment in argon was used to modify the surface for 5 min. After plasma activation, the sample was exposed to the atmosphere for 20 min, and then immersed in 1 M of a sodium hydroxide (NaOH) solution for 24 h. The TiO_2_ nanoparticles were prepared as follows:

- Solution A: 5 wt % of titanium isopropoxide (Sigma-Aldrich, Sigma-Aldrich Sp. Z.o.o., Poznan, Poland) was mixed in a propanol solvent at 50 °C.
- Solution B: 5 wt % of diluted acetic acid was prepared.
- Solution C: Solution B was slowly poured into solution A at a ratio of 50:50 *v*/*v*.
- Solution D: Solution C was heated to remove any water.
- Solution E: Sodium hydroxide (NaOH) was used to neutralize the pH of solution D.

After the described procedure, the titanium dioxide with some amount of aliphatic chains was obtained. The presence of oxygen groups into the aliphatic chains was confirmed by FTIR analysis, shown in [39]. Hence, the crystallinity structure of TiO_2_ can be classified as a polyamorous. Therefore, the average particle size of these materials will be larger than for pure anatase or rutile structure of TiO_2_. However, application of acetic acid provided a decrease of average particle size of TiO_2_ [40]. To show the photoactivity of the titanium dioxide layer, the photodegradation examination with BSA (bovine serum albumin) fouled layers were performed. These results are widely explained in our paper [38].

Finally, the membranes, after plasma and chemical treatments, were immersed into solution E for 2 days. The samples were rinsed and kept in distilled water.

PVDF/PAN 1/2 nanofibrous membranes were treated by plasma and chemical methods in four different configurations, as shown in Table 1.

It was proven that the TiO_2_ surface becomes more hydrophilic after UV irradiation [41,42]. In this study, the effect of the UV irradiation on the fouling of TiO_2_ covered nanofibrous membranes has been investigated.

### 2.4. Filtration and Self-Cleaning Experiments

**Figure 3:**
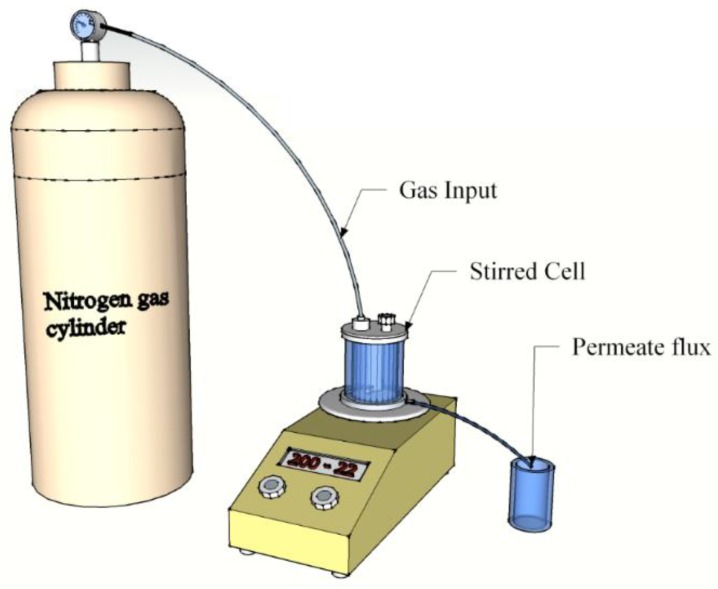
Schematic diagram of dead-end filtration.

The oil–water separation was carried out with a 50 mL Millipore Amicon stirred filtration cell (Millipore Corporation Billerica, MA, USA). A schematic diagram of the dead-end device is shown in Figure 3. The oil–water mixture (50%, v/v) was poured into the device. The water was coloured by methylene blue to properly observe the separation process. The feed solution was mixed with a hand mixer for a few minutes, until a uniform mixture was obtained. Subsequently, 90 mL of distilled water was filtrated, followed by 45 mL of the oil–water mixture. This procedure was repeated a few times to determine the anti-fouling properties of the membrane. The membrane was not changed or replaced during each repeating step. The separation process was performed under a 0.02 bar pressure.

The permeate flux (*F*) and the permeability (*k*) of the membrane were calculated (Equations (1) and (2)):

(1) $$F=\frac{1}{A}\frac{dV}{dt}$$

(2) $$k=\frac{F}{p}$$

where *A* is the effective membrane area (m^2^), *V* is the total volume of the permeate (*F*), *p* is the transmembrane pressure (bar), and *t* is the filtration time [43].

## 3. Results

### 3.1. Membrane Characterization

**Figure 4:**
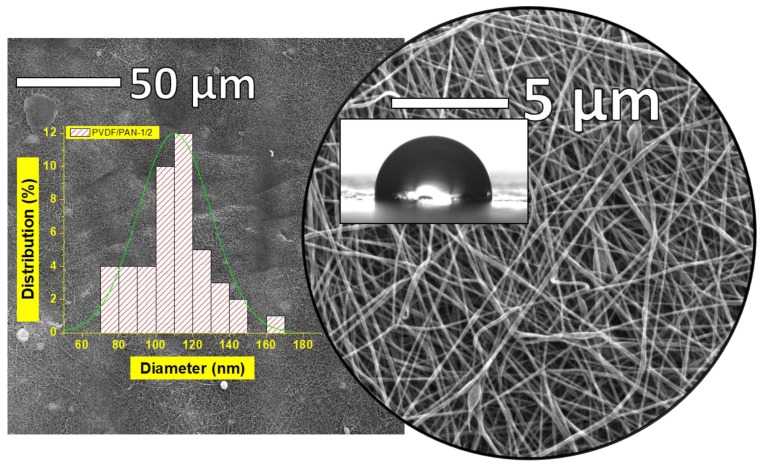
SEM images of PVDF/PAN nanofibers after the lamination process.

The SEM images are given in Figure 4. The SEM images demonstrated that the lamination process did not damage the surface of the nanofiber layer. However, there were some blind spots, where the adhesive web covered the surface of the nanofibers and totally blocked the pores. In the resultant web, blind spots were rarely observed. It was not possible to remove these blind spots without delaminating the membranes, but it was possible to keep their number as low as possible. The fiber diameter of the nanofiber was around 110 nm, which was good for the filtration process, due to the small pore size. The pore size of the nanofiber was related to the diameter of the fiber. A lower fiber diameter yielded a lower mean pore size.

Table 2 shows the characterization of the nanofiber layer. The basis weight of the nanofiber web was less than 1 g/m^2^, which was advantageous at high production speeds, and led to low production costs. The porosity of the membrane was quite important for the permeability of the membranes. In this study, the nanofiber layer had a porosity of more than 85% of its bulk volume. In theory, the low water contact angle indicates higher hydrophilicity and better wettability that increases the water permeability through the membrane. It is well know that hydrophilic membranes decrease the fouling due to the high affinity of the membrane to water molecules [44]. The pristine PVDF/PAN membrane without any surface treatment can be considered as being “hydrophobic” by definition [43].

As shown in Table 2, the water contact angle of the pristine PVDF/PAN membranes was 92.7°. After surface modification with plasma, the water contact angle of the membranes decreased to 0°. These results indicate that the hydrophilicity of the membranes was improved by plasma and chemical modification.

**Figure 5:**
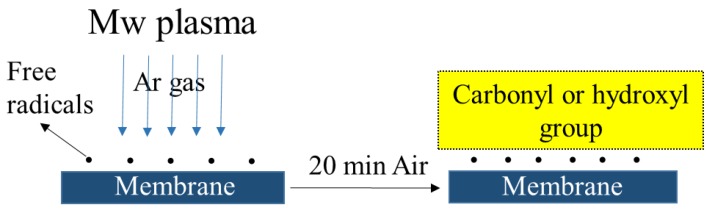
Functionalization of membranes by atmospheric exposure subsequent to Ar plasma treatment.

All the surface modified membranes showed a “zero” water contact angle. The surface wettability of the membranes was improved by the plasma and plasma + chemical modification, due to the introduction of hydroxyl groups. Clouet et al. observed that argon plasma can be used to introduce oxygen functionality into the surface of the material [45]. For inert Ar gas plasma, functionalization of the surface is thought to take place on atmospheric exposure after the plasma treatment, as shown in Figure 5.

The carboxyl/hydroxyl groups attached to the surface of the membrane increases the hydrophilicity of the membranes.

**Figure 6:**
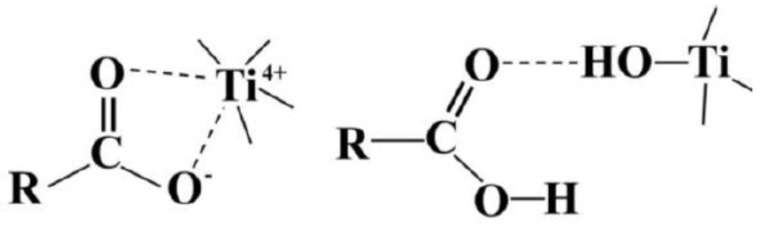
Mechanisms of self-assembly of TiO_2_ with a polymer surface. Reproduction from [46].

After incorporation, carboxyl or hydroxyl groups onto the fiber surface titanium dioxide deposition was performed. Particles of TiO_2_ with aliphatic chains containing oxygen were obtained via sol-gel method. The mechanism of self-assembly of TiO_2_ on the polymer surface is described in Figure 6.

In the first case, the titanium particles are connected to the surface by the ether coordination bonds, while in the second, the Ti is bonded by hydroxyl groups to the alkyl moieties. To verify the photoactivity of TiO_2_ on the polymer surface, the photodegradation process was conducted. The results showed that the fouled layer of BSA could be removed from the membrane surface with 95% efficiency after UV irradiation in the presence of TiO_2_ particles [38]. Unfortunately, the TiO_2_ particles on the surface were not visible by means of our SEM instrument. We were only able to determine that they did not aggregate.

It was found that NaOH-induced hydrolysis of nitrile groups on the PAN surface resulted in increasing of membranes swelling with the time of treatment [47]. Yang et al. [48] hydrolyzed PAN hollow fiber in different concentrations of NaOH solution (0.5, 1 and 2 N). By increasing the concentration of NaOH, the concentration of carboxylic groups greatly increased. However, the highest concentration caused severe degradation of PAN fibers. In another paper dealing with chemical modification with NaOH, the decrease of water flux during the progress of hydrolysis with the increase of solute rejection was observed [49].

### 3.2. Filtration and Self-Cleaning Experiments

**Figure 7:**
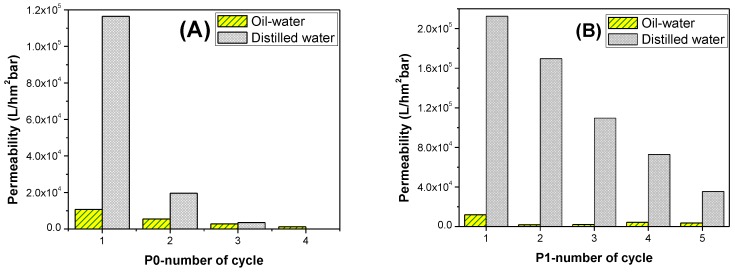

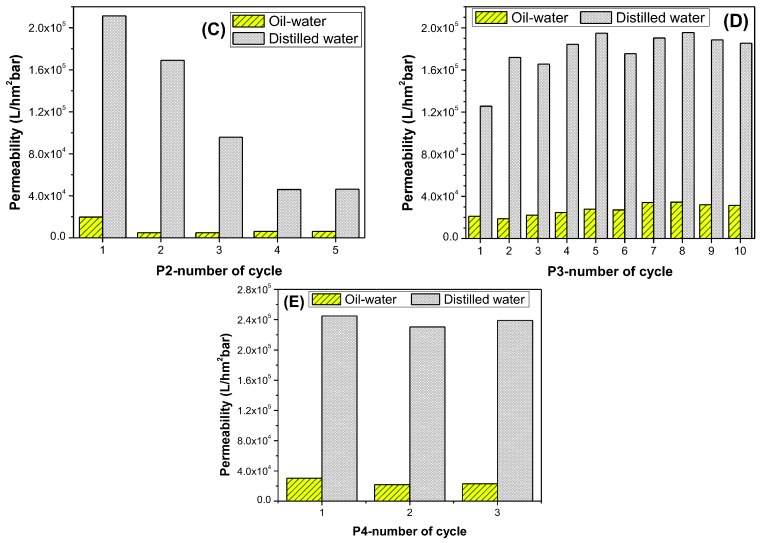
Repeated fouling and self-cleaning experiments of samples (**A**) P0, (**B**) P1, (**C**) P2, (**D**) P3, and (**E**) P4.

In this study, oil/water separation experiments were conducted by using a dead-end filtration device. The permeability of the each membrane was calculated according to Equation (2). Figure 7 shows the permeability of membranes for alternated filtration of water and the oily water. The process was repeated in each cycle. Between each cycle, the membranes were washed gently.

The performance of the membranes without post treatment is shown in Figure 7A. In each cycle, the filtration efficiency and the permeability of the membranes decreased drastically due to the fouling phenomenon. The permeability of the membranes with pure water decreased over 3000 times from the first to the fourth cycle. The same filtration protocol was repeated for the treated membranes.

**Figure 8:**
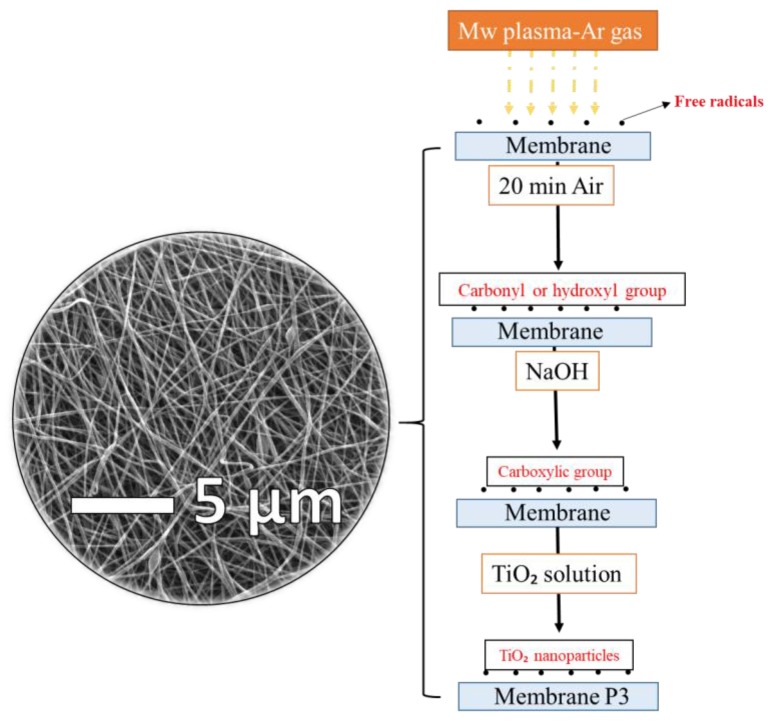
Schematic diagram of the surface modification of PVDF/PAN membranes.

Membrane P1 showed better permeability than P0, due to the increase of hydrophilicity after treatment. However, fouling was inevitable. At the end of the fifth cycle, the pure water permeability of the membranes decreased 6 times. A similar trend was observed for membrane P2. Immersing the membrane into the TiO_2_ solution after plasma treatment did not improve the membrane performance. On the other hand, membrane P3 showed excellent permeability with antifouling properties, even after the tenth cycle. A mid-treatment between plasma and TiO_2_ modification was necessary. Immersion in NaOH solution is an effective method for grafting TiO_2_ to the surface of the membrane, due to the creation of carboxylic groups. Once the sufficient surface grafting of TiO_2_ had been obtained, the membrane fouling-resistance and membrane permeability improved. A schematic diagram of the chemical modification process is shown in Figure 8.

In the first stage, membranes treated with Ar microwave plasma were exposed to air for 20 min. Carbonyl/hydroxyl groups were formed on the surface of the membranes. Formation of carboxylic groups on the surface occurred after NaOH treatment. Eventually, TiO_2_ particles were grafted to the activated surface.

It is well known that UV radiation activates TiO_2_ located on the surface. For this aim, membrane P4 was prepared and tested on a filtration unit. The results show that there is no high permeability difference between membranes P3 and P4. As UV treatment generates an extra cost, it can be concluded that such operation is not needed to improve membrane permeability.

**Figure 9:**
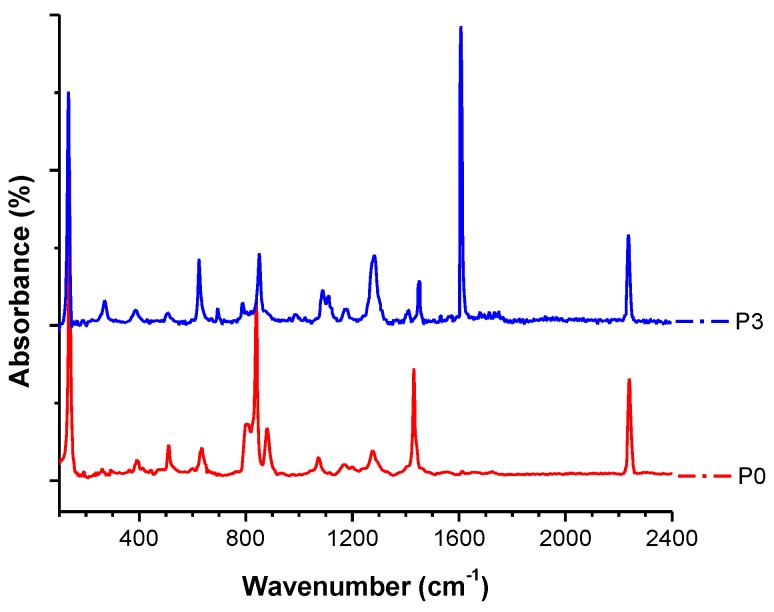
FTIR spectra of the neat sample and the sample after modification.

The FTIR spectra were collected in order to investigate the chemical structure of the pristine membrane, and the membrane after plasma and chemical modification. Figure 9 confirms the presence of both polymers in the blend of PVDF/PAN. Stretching bands at 1175 cm^−1^, 1412 cm^−1^, and 876 cm^−1^ for the –CF_2_ and C–F groups of PVDF, and absorption bands at 2239 cm^−1^ for the PAN nitrile groups, were observed. The TiO_2_ sample had transmittance peaks in the range of 500–1000 cm^−1^, which was assigned to the vibrations of Ti–O and Ti–O–Ti framework bonds. The bands around 1619 cm^−1^ corresponded to the bending modes of water Ti–OH [50,51,52,53].

**Figure 10:**
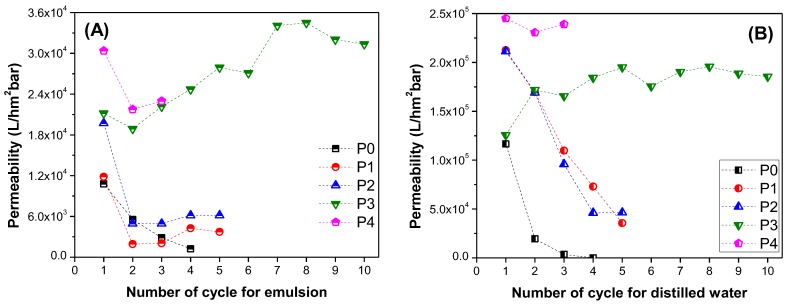
Permeability vs the number of cycles for (**A**) oil–water, and (**B**) distilled water.

Figure 10 showed the permeability comparison of all of the membranes. The oily water and the pure water permeability was compared separately. All of the surface modified membranes exhibited higher pure water permeability than the pristine membrane. Moreover, membrane P3 has higher permeability and better anti-fouling properties compared to P0, P1, and P2, and its fouling resistance property were remarkably improved. It can be concluded that the TiO_2_ grafted to the hydrophilic membrane surface prevented direct adhesion of the oil droplets. Chen et al. [54] prepared a porous PVDF–MWCNT (multiwalled carbon nanotube) foam, which was characterized as a reusable and compressible superhydrophobic–superoleophilic separator with good elasticity and low surface energy. The porous PVDF–MWCNT foam exhibited high adsorption capacity to a variety of oils/organic solvents that made it a promising candidate for large-scale industrial applications. Unlike our membranes, their foams worked on the adsorption principle with a capacity 300–1200% of its own weight. Our membranes P0–P4 worked in the permeability principle, and did not need any additional treatment.

**Figure 11:**
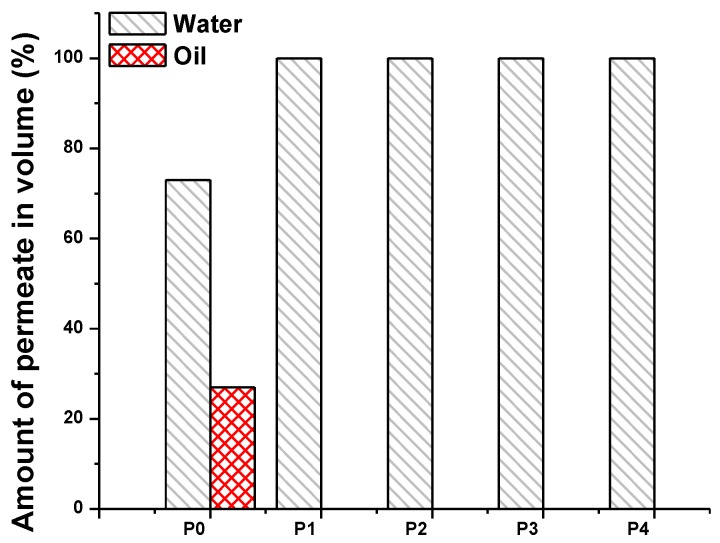
Selectivity of the membranes P0–P4.

The selectivity of the membranes is shown in Figure 11. The feed solution after separation was collected, and the volume ratio of the oil and water was measured. The percentage of the selected liquid was calculated as follows:

(3) $$amount\;of\;selected\;liquid=\frac{V_{selected\;liquid}}{V_{total\;feed}}\times 100\;\left(\%\right)$$

*V* is the volume in liters.

Figure 11 shows that the neat PVDF/PAN membrane is both hydrophilic and oleophilic, while the surface treated membranes are more hydrophilic. The water selectivity of the pristine PVDF/PAN membrane improves from 73 to 100% as soon as the surface modification took place. The oleophobic characteristics of the membranes made them attractive for the filtration of oily wastewater. Even though the membranes showed good hydrophilicity and oleophobicity, they were not sufficient enough to be applied to the separation process. The sought membranes should be easily cleanable, and should not lose their performance over time. Only two of the investigated membranes, P3 and P4, fulfill the properties of ideal membranes for oil–water separation. On the other hand, the permeability of membranes P3 and P4 was almost the same for the separation of oily wastewater. We determined that membrane P3 was the best membrane for separation, and that there was no need to expose it to UV light in order to activate the TiO_2_ particles on the membrane surface. The method used for the surface modification proved that highly permeable and highly selective membranes can be obtained for the separation of oily wastewater.

## 4. Conclusions

In summary, a PVDF/PAN nanofiber web was successfully fabricated and laminated. The resultant membranes showed both hydrophilic and oleophilic characteristics. A series of surface modifications were applied to the PVDF/PAN membranes to enhance the hydrophilicity and permeability of the membranes.

It was found that Ar-plasma surface treatment was not multifunctional; therefore, more than one chemical modification was required to accommodate the grafting of a functional TiO_2_ group onto the membrane surface. Since the surface of the TiO_2_-grafted membranes was able to build a highly hydrophilic and low surface energy barrier against the adhesion of oil droplets, the permeability and the antifouling properties were significantly enhanced. Undoubtedly, the most important part of the surface modification technique was grafting of TiO_2_ onto the surface of the membrane.

In conclusion, we have reported a facile and low-cost method for the preparation of hydrophilic/oleophobic membranes by using a new surface modification approach with a plasma and chemical method. Needless to say, surface treated polymeric PVDF/PAN membranes are a good candidate for use in separation technologies for water/oil emulsions.

## Figures and Tables

**Table 1:** Preparation of nanofibrous composite membranes in various ways for different combinations of plasma and chemical methods.

Abbreviation of the Sample	Plasma Modification	Chemical Modification	UV Irradiation
P0*	-	-	-
P1	5 min Plasma + 20 min exposed to atmosphere	Immersed into NaOH for 24 h	-
P2	5 min Plasma + 20 min exposed to atmosphere	Immersed into solution E for 2 days	-
P3	5 min Plasma + 20 min exposed to atmosphere	Immersed into NaOH for 24 h and solution E for 2 days	-
P4	5 min Plasma + 20 min exposed to atmosphere	Immersed into NaOH for 24 h and solution E for 2 days	4 min under UV light

P0* is a neat PVDF/PAN membrane without any post-treatment.

**Table 2:** Membrane characterization for PVDF/PAN nanofibers.

Polymer	Viscosity (Pa.s)	Basis Weight of Nanofiber (g/m^2^)	Fiber Diameter (nm)	Porosity (%)	Avr. Pore Size (nm)	Contact Angle (°)
PVDF/PAN	0.35	0.76 ± 0.50	110.18 ± 19.90	>85	820 ± 32	92.7 ± 3
